# Supraclavicular Solitary Hybrid Schwannoma/Neurofibroma: A Case Report

**DOI:** 10.7759/cureus.8531

**Published:** 2020-06-09

**Authors:** Alanoud Alomair, Mohammad Dababo, Suresh Velagapudi

**Affiliations:** 1 Otolaryngology, King Faisal Specialist Hospital and Research Centre, Riyadh, SAU; 2 Anatomic Pathology, King Faisal Specialist Hospital and Research Centre, Riyadh, SAU

**Keywords:** schwannoma, neurofibroma, hybrid

## Abstract

Peripheral nerve sheath tumors (PNSTs) are benign lesions arising from the connective tissue sheath surrounding the neurons and are labeled schwannoma, perineurioma, or neurofibroma according to their histopathological characteristics. Lesions with a mixture of two or more of the aforementioned tumors are known as hybrid peripheral nerve sheath tumors (HPNSTs). These hybrid tumors have been described as rare entities. In this report, we present a case of a solitary hybrid schwannoma/neurofibroma in an unusual location.

## Introduction

Peripheral nerve sheath tumors (PNST) are benign lesions arising from the connective tissue sheath surrounding the neurons. Most of these tumors present as focal soft tissue swelling with symptoms attributable to mass effect to adjacent structures. Diagnostic classifications based on the histopathology of PNST have been published previously [1]. The most common of these are schwannoma, perineurioma, and neurofibroma. A lesion with histopathological evidence of a nerve sheath tumor not specific to one of the aforementioned tumors is classified as a hybrid peripheral nerve sheath tumor (HPNST). These tumors are a benign combination with shared characteristics of the previously mentioned tumors. HPNSTs were officially introduced in the fourth edition of the World Health Organization (WHO) Classification of Tumors of Soft Tissue and Bone published in 2013, with the revised edition being released in 2016 [2,3]. Combinations occur either sporadically, accounting for schwannoma/perineurioma, or with associated syndromes such as neurofibromatosis (NF), which includes schwannoma/neurofibroma [2,4,5].

## Case presentation

A 35-year-old male, a known case of type I autosomal dominant cerebellar ataxia, was referred to our otolaryngology clinic for a slowly progressing neck mass for three years. Upon further questioning, the patient had no associated symptoms or significant family history. Examination of the swelling yielded a palpable, non-tender mobile mass in the left supraclavicular region. The left upper limb had no sensory or motor deficits. The rest of the examination was insignificant. The patient showed no signs of stigmata of NF or schwannomatosis.

**Figure 1:**
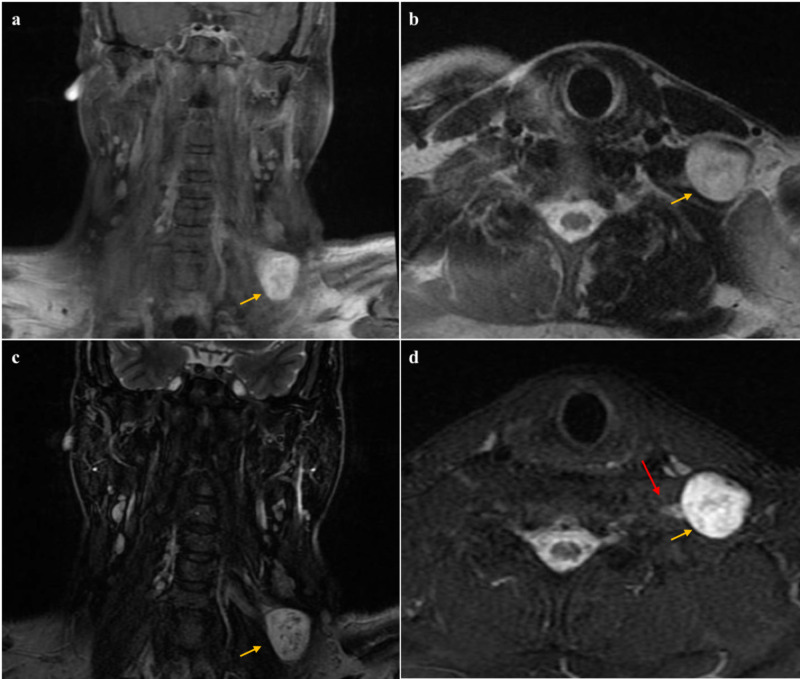
MRI images of the patient's neck The yellow arrow indicates the tumor; a and b: T1-weighted images, coronal and axial respectively, showing heterogenous hyperintensity of the tumor; c and d: T2-weighted images, coronal and axial, showing hyperintensity of the tumor and increased intensity of C6 nerve root (red arrow) MRI: magnetic resonance imaging

The patient was sent for a neck MRI, which showed a left lower supraclavicular, well-circumscribed, heterogeneous hyperintense enhancing mass measuring 30 x 26 mm (Figure 1). The lesion was located posteromedial to the sternocleidomastoid muscle and abutting the C6 nerve root.

**Figure 2:**
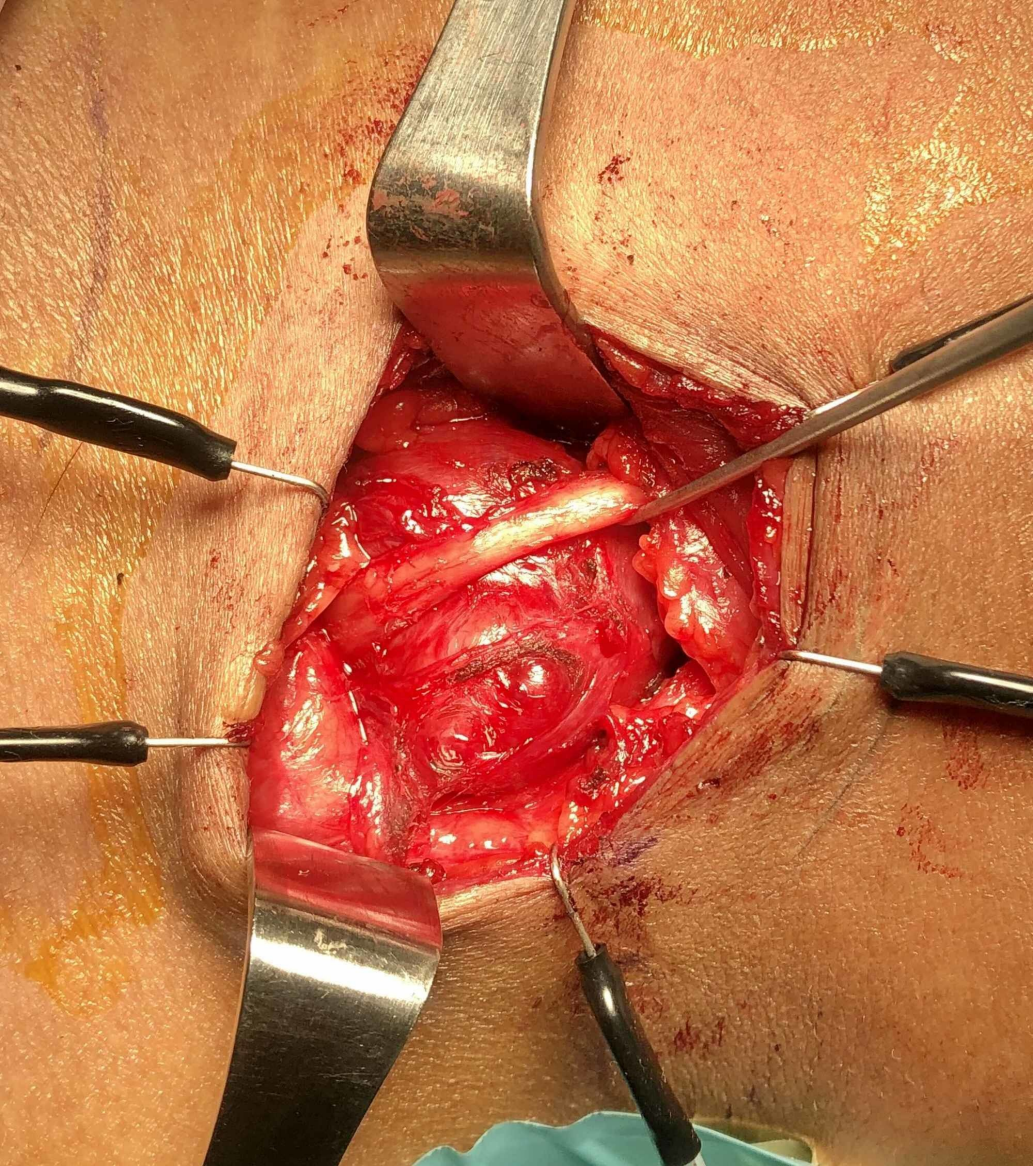
Intraoperative picture of the nerve sheath tumor prior to resection with overlying brachial plexus

Core biopsy of the mass was taken, and histopathology was well consistent with schwannoma. The lesion was excised surgically through an anterior supraclavicular approach. The brachial plexus was identified intraoperatively and preserved (Figure 2). On gross inspection, the tumor was pale, encapsulated, and measured 30 mm in its greatest dimension.

**Figure 3:**
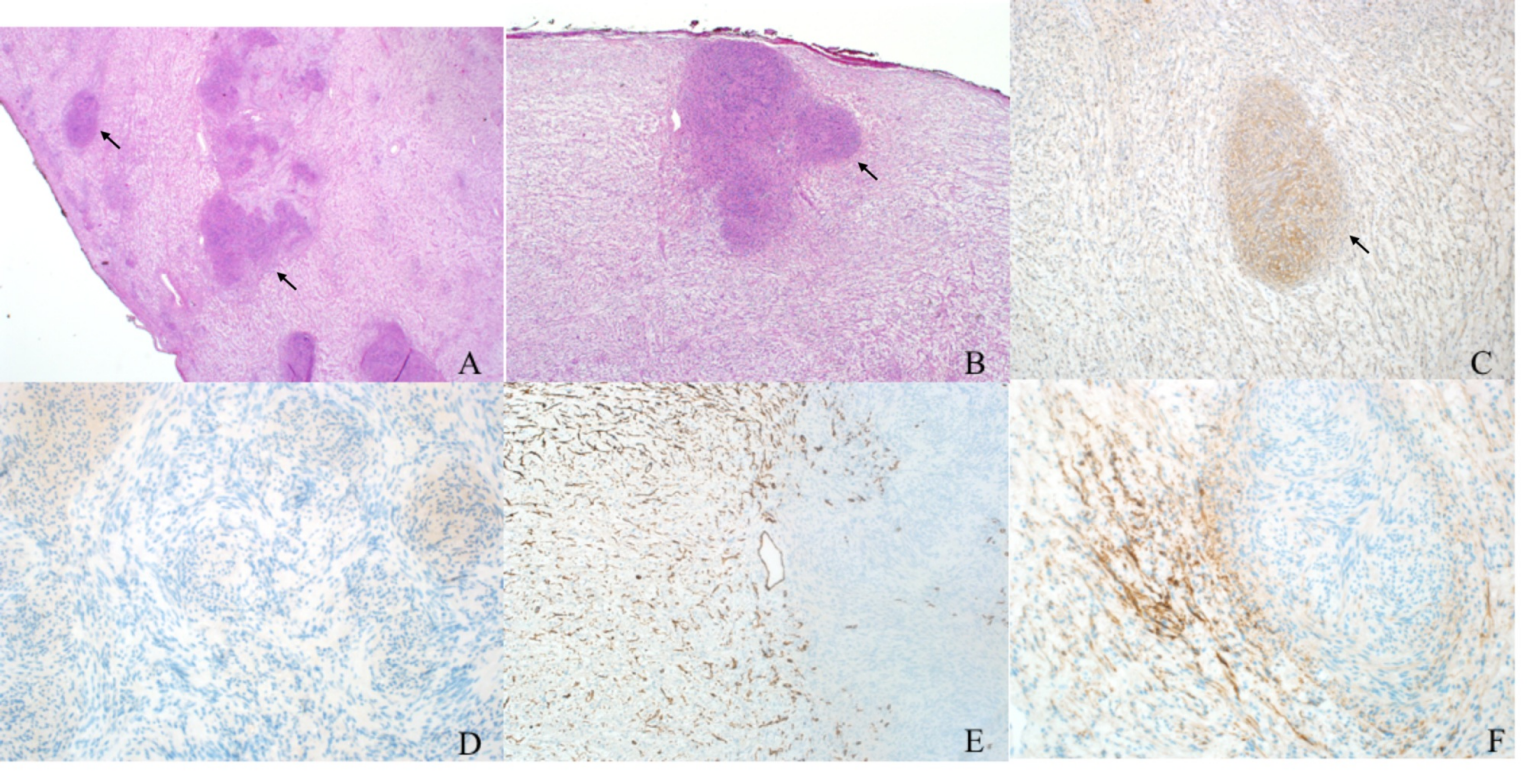
Micrographs of the tumor A and B: low-power images of H&E stain showing well-defined nodules of schwannoma (arrows) within a background of neurofibroma. C: S100 staining of the schwannoma (arrow) compared to the neurofibroma area. D: NFP staining showing the absence of the axons in the schwannoma nodules. E: CD34 staining with higher positivity in the blood vessel wall as well as the neurofibroma component. F: GFAP staining of the neurofibroma component H&E: hematoxylin and eosin; NFP: neurofilament protein; GFAP: glial fibrillary acidic protein

Histopathologically, schwannoma nodules showed areas of Antoni A and Antoni B, which is different from the neurofibroma component. Multiple immunohistochemical stains were tested to confirm the diagnosis, including S100, which showed higher positivity in the schwannoma component compared to the neurofibroma. Neurofilament protein (NFP) showed entrapped axons in the neurofibroma component, while in the schwannoma components, axons were absent. Both CD34 and glial fibrillary acidic protein (GFAP) stains showed positivity in the neurofibroma component only (Figure 3).

Postoperatively, the patient was not able to abduct his left shoulder beyond 90 degrees. Nerve conduction study (electromyography) suggested mild hyperacute upper trunk brachial plexopathy. However, at the two-month follow-up visit, the patient was able to fully abduct his shoulder. At the six-month follow-up, the patient showed no evidence of tumor recurrence.

## Discussion

PNSTs were classically divided into three categories: schwannoma, neurofibroma, and perineurioma [6]. In recent years, a new entity of soft tissue tumor known as hybrid peripheral sheath tumor was introduced by the World Health Organization, in which two discrete histology are evident within the same mass [3]. Histopathologically, schwannoma is consistent with hypercellular areas with spindle cells arranged in fascicles (Antoni A) and myxoid areas with low cellularity (Antoni B), whereas neurofibroma shows axons, Schwann, perineural, and inflammatory cells on a background of collagen [7]. In these hybrid tumors, we see a mixture of schwannian nodules in a background of neurofibromatous component.

Unfortunately, solitary hybrid schwannoma/neurofibroma tumors have been rarely reported in the literature. Such cases were initially described by Feany et al. in a series of nine cases. Three out of the nine reported cases failed to show signs or features of NF, suggesting that hybrid tumors are not confined to NF alone [5]. Only one of the cases had a recurrence.

Later on, multiple cases were reported in the literature in English by different authors representing solitary hybrid schwannoma/neurofibroma in different locations (Table 1). All those cases failed to show clinical stigmata of NF or schwannomatosis [2,6-11].

**Table 1:** Reported cases of solitary hybrid schwannoma/neurofibroma NSR: no signs of recurrence; NA: not available

Case no	Reference	Year	Age at presentation	Sex	Site	Size (mm)	Follow-up
1	Murărescu et al. [6]	2005	20	M	Chest wall	40	NA
2	Youens et al. [8]	2008	51	F	Orbit	21	NA
3	Panda et al. [9]	2015	30	M	Scalp	40	NSR, 6 months
4	Hussain et al. [10]	2016	47	M	Hip	14	NA
5	Shanouda et al. [11]	2017	24	M	Ankle		NSR, 8 months
6	Taubenslag et al. [7]	2017	31	M	Orbit	34	NA
7	Ud Din et al. [2]	2017	5	M	Thigh	55	NA
8	Present case	2019	35	M	Neck	30	NSR, 6 months

The development of schwannian differentiation within a neurofibroma remains unclear. It has been suggested to be due to clonal genetic alteration in the microenvironment [5]. Nevertheless, no malignant transformation of hybrid schwannoma/neurofibroma has been reported in the literature. Harder et al. has reported the presence of hybrid schwannoma/neurofibroma in 23 patients, which were found in association with NF type1, type 2, and schwannomatosis [12]. Unlike our present case, the majority of them were plexiform.

## Conclusions

This report described a rare entity of PNST that might be diagnostically challenging or can even go underdiagnosed. The absence of NF or schwannomatosis stigmata makes the diagnosis even more difficult. However, advances in molecular testing have shown an increase in the number of reported cases. To date, the exact etiological factors remain unclear. In conclusion, schwannoma/neurofibroma tumors resemble benign peripheral nerve sheath pathology with no reported malignant transformation reported in the literature so far.
